# Unveiling CRESS DNA Virus Diversity in Oysters by Virome

**DOI:** 10.3390/v16020228

**Published:** 2024-01-31

**Authors:** Peng Zhu, Chang Liu, Guang-Feng Liu, Hong Liu, Ke-Ming Xie, Hong-Sai Zhang, Xin Xu, Jian Xiao, Jing-Zhe Jiang

**Affiliations:** 1 College of Oceanography and Ecological Science, Shanghai Ocean University, Shanghai 201306, China; 2 Key Laboratory of South China Sea Fishery Resources Exploitation and Utilization, Ministry of Agriculture and Rural Affairs, South China Sea Fisheries Research Institute, Chinese Academy of Fishery Sciences, Guangzhou 510000, China; 3 Animal and Plant Inspection and Quarantine Technology Centre, Shenzhen Customs, Shenzhen 518000, China; 4 Southern Marine Science and Engineering Guangdong Laboratory (Zhuhai), School of Marine Sciences, Sun Yat-sen University, Zhuhai 519000, China; 5 School of Life Science and Biopharmacy, Guangdong Pharmaceutical University, Guangzhou 510000, China; 6 Livestock, Aquaculture and Technology Promotion and Service Center of Conghua District, Guangzhou 510000, China

**Keywords:** oyster, virome, CRESS DNA virus, phylogenetic tree, Rep, Cap

## Abstract

Oysters that filter feed can accumulate numerous pathogens, including viruses, which can serve as a valuable viral repository. As oyster farming becomes more prevalent, concerns are mounting about diseases that can harm both cultivated and wild oysters. Unfortunately, there is a lack of research on the viruses and other factors that can cause illness in shellfish. This means that it is harder to find ways to prevent these diseases and protect the oysters. This is part of a previously started project, the Dataset of Oyster Virome, in which we further study 30 almost complete genomes of oyster-associated CRESS DNA viruses. The replication-associated proteins and capsid proteins found in CRESS DNA viruses display varying evolutionary rates and frequently undergo recombination. Additionally, some CRESS DNA viruses have the capability for cross-species transmission. A plethora of unclassified CRESS DNA viruses are detectable in transcriptome libraries, exhibiting higher levels of transcriptional activity than those found in metagenome libraries. The study significantly enhances our understanding of the diversity of oyster-associated CRESS DNA viruses, emphasizing the widespread presence of CRESS DNA viruses in the natural environment and the substantial portion of CRESS DNA viruses that remain unidentified. This study’s findings provide a basis for further research on the biological and ecological roles of viruses in oysters and their environment.

## 1. Introduction

Belonging to the class *Bivalvia*, family *Ostreidae*, oysters are filter-feeding organisms that can be found in intertidal zones worldwide. Oysters can filter up to five liters of seawater every hour through their gills, enriching suspended microorganisms and particles by a factor of 10,000 to 100,000 times their seawater concentrations. This makes them excellent accumulators of viral particles as well. Oysters play a crucial role in the ecology of coastal and estuarine ecosystems due to their filter-feeding behaviors and sessile lifestyle, providing a stable and lasting environment for various organisms to thrive [1,2,3].

As oysters lack an acquired immune system and lead a stationary existence in clusters, the likelihood of viral transmission within their population may be heightened [4]. Early on, using classical electron microscopy techniques, various viral pathogens were observed in oysters, including ostreid herpesvirus-1 (OsHV-1) (family *Malacoherpesviridae*); gill necrosis virus (GNV) (family *Iridoviridae*); and putative representatives of other families, including *Papovaviridae*, *Togaviridae*, *Reoviridae*, and *Picornaviridae* [5,6,7]. Owing to the proximity of oyster habitats to coastal regions affected by anthropogenic activities, oysters tend to accumulate pathogenic viruses that can pose a significant threat to human health. Some of the commonly found viruses in oysters include norovirus (NV), hepatitis A virus (HAV), and astrovirus (AV) [8]. In recent years, researchers have employed high-throughput sequencing techniques to identify 33 novel RNA viruses from mixed bivalve samples comprising *Crassostrea hongkongensis* and *Crassostrea ariakensis*. These viruses have been classified into various families, including *Narnaviridae*, Yanvirus, Weivirus, *Totiviridae*, *Tombusviridae*, *Picornavirales*, and *Nodaviridae*. The discovery of these novel RNA viruses has significantly contributed to understanding the viral diversity and evolution in bivalve populations [9]. Rosani et al. conducted a comprehensive analysis of publicly available transcriptome data of *Crassostrea gigas* and *Crassostrea corteziensis* to assemble 26 new RNA virus genomes. The identified viruses predominantly belonged to the orders *Picornavirales* (families *Dicistroviridae* and *Marnaviridae*) and *Herpesvirales* (family *Malacoherpesviridae*) [10,11,12]. An initial study by Jiang et al. reported the Dataset of Oyster Virome (DOV), containing 3473 meticulously curated viral genomes. Among them, there are at least 11 DNA virus families present, which comprise bacteriophages and CRESS DNA viruses mainly. Additionally, 18 new RNA viruses belonging to five distinct virus orders or families (*Sobelivirales*, *Picornavirales*, *Durnavirales*, *Leviviridae*, and Yanvirus) were identified [13,14].

CRESS (eukaryotic circular rep-encoding single-stranded) DNA viruses are a group of circular single-stranded DNA viruses with genome sizes ranging from 1.7 kb to 3 kb and containing only 2–6 protein-encoding genes [15]. According to the most recent classification system established by the International Committee on Taxonomy of Viruses (ICTV), *Cressdnaviricota* is represented by two classes, eight orders, and 12 families (*Bacilladnaviridae*, *Circoviridae*, *Geminiviridae*, *Genomoviridae*, *Metaxyviridae*, *Amesuviridae*, *Naryaviridae*, *Nanoviridae*, *Nenyaviridae*, *Redondoviridae*, *Smacoviridae*, and *Vilyaviridae*) [16,17]. *Cressdnaviricota* includes many pathogenic taxa that infect eukaryotes, which have a serious impact on human production and livelihoods. For example, *Circoviridae* and *Geminiviridae* can infect animals and cause some diseases. *Nanoviridae* and *Metaxyviridae* are the two largest groups within the phylum *Cressdnaviricota* and are known to be pathogens of various economically important crops. These viral agents have been identified as the cause of global crop yield reduction, leading to significant economic losses for farmers and agricultural industries worldwide [18]. *Redondoviridae* is a recently identified group of CRESS DNA viruses that have been detected in the human respiratory tract. These viruses are closely linked to several pathological conditions, including periodontitis and other diseases [19].

Dayaram and Gezon et al. found the presence of large numbers of CRESS DNA viruses in estuarine mollusks, such as *Austrovenus stutchburyi*, *Paphies subtriangulata*, *Dreissena rostriformis bugensis*, and *Amphibola crenata*, and bottom sediments. These CRESS DNA viruses seem to be ubiquitous and very diverse [20,21]. A significant finding of DOV is the identification of numerous unclassified CRESS DNA virus-related sequences (both complete genomes and genome fragments) in a single species, *Crassostrea hongkongensis* [13,22]. A total of 8763 complete replication-associated protein (Rep) sequences were further discovered, among which 19 Reps are closely related to *Circovirus* and *Cyclovirus*, two genuses in the *Circoviridae* family. According to the phylogenetic relationship, 19 Reps were tentatively named Crasscircovirus [13]. The report of DOV highlights the rich diversity of CRESS DNA viruses in oysters, but it lacks in-depth analysis of the complete genome of these novel viruses. It is also still unclear about the relationship between the DOV viruses and their relatives classified in *Cressdnaviricota* as well as their potential harm to oysters. Given this, this study delves into the in-depth examination of the 30 complete genomes of CRESS DNA viruses from DOV. This effort will shed light on the classification, diversity, evolution, and identification of viral pathogens in oysters within the CRESS DNA viruses.

## 2. Materials and Methods

### 2.1. Sequence Assembling and Virus Discovery

In the previous phase of the DOV project [13], metagenomic and metatranscriptomic libraries were constructed with oyster samples collected from 2014 to 2019 from 9 locations (Qinzhou area of Guangxi Province, Huidong, Huangsha, Shenzhen, Taishan, Tanwei, Zhuhai, Yangjiang, and Lianjiang areas of Guangdong Province) along the southern coast of China. Finally, 54 virome libraries were sequenced, resulting in approximately 2.5 billion raw sequencing reads [13]. The sequencing data were processed using Fastp (version 0.20.0) [23]. The reads were then assembled into contigs using MEGAHIT (version 1.2.9) [24,25]. Contigs were aligned and annotated with the National Center for Biotechnology Information (NCBI) nonredundant protein (NR) database using DIAMOND (version 0.9.24.125) [26]. We classified the annotated sequences using MEGAN6 [27]. Finally, 30 genomes initially identified as CRESS DNA viral genomes were selected for further analysis. As the genomes are circular, a genome is considered complete if an overlap exists at both ends of the genome. If not, the genome is incomplete.

### 2.2. Open Reading Frame (ORF) Prediction and Annotation

ORFs were predicted in the 30 viral genomes using Prodigal [28], and the completeness of ORFs was determined based on the partial value of protein sequences output by Prodigal. The ORF sequences were aligned to the NR database using NCBI BLASTP [29,30] with an e-value cutoff set to 10^−5^. The protein sequences with the highest consistency were then aligned inversely with the virus genome sequences using NCBI tBLASTN to ensure the accuracy of the ORF predictions. The genome structure was visualized using SnapGene (version 4.3.6).

### 2.3. Similarity Clustering Analysis

We conducted Blastp comparisons in the NCBI NR database to obtain the top ten protein sequences based on total score and constructed Rep and capsid proteins (Cap) databases separately using the Rep sequences and Cap sequences from *Cressdnaviricota* (12 families) identified by ICTV, Crasscircoviruses reported by Jiang et al., and CRESS DNA viruses reported by Kazlauskas [31]. DIAMOND was employed for sequence alignment. Then, we used Gephi (version 0.9.2) [32] to construct clustering networks based on the scores. We performed a genome average nucleotide identity heatmap by the Sequence Demarcation Tool (SDT) (version 1.3) [33], including genomes of 30 CRESS DNA viruses in this study and corresponding genomes of Rep and Cap in the NR database, which are closely related to proteins of 30 CREAA DNA viruses in this study. When SDT was successful, MAFFT was used for alignment.

### 2.4. Phylogenetic Tree Construction Based on Rep and Cap

We used MAFFT [34] for multiple sequence alignment. TrimAL [35] was used to remove poorly aligned regions from alignment results. Maximum likelihood phylogenetic trees were built by IQtree (version 2.1.4) [36] based on the amino acid sequences of Rep and Cap. ModelFinder [37] was set to MFP (for ModelFinder Plus), and 1000 ultrafast bootstrap replicates were utilized. Finally, visualization was accomplished through iTOL (version 6.5.2) [38] (https://itol.embl.de) (accessed on 12 October 2023).

### 2.5. Analysis of the Abundance of Viruses

To determine the relative abundance of each virus, we combined 30 viral genome sequences and created a reference genome dataset using the Salmon (version 0.13.1) [39] index command. Then, we utilized the Salmon Quant command to map the clean reads of all the oyster virome libraries (PRJCA007058) individually to the reference genome. Finally, we counted the number of mapped reads for each library and calculated the relative abundance values of each virus using the adjusted TPM (transcripts per million) calculation formula.

$$TPM=\frac{Genome\;reads}{Genome\;length}$$

where genome reads is the number of mapped reads, genome length is the length of the genome in Kb, and total TPM represents the sum of the TPM values from individual libraries.

## 3. Results

### 3.1. Ubiquitous and Abundant Oyster-Associated CRESS DNA Viruses

Based on the previous research conducted by Jiang et al. [13], we selected 30 fully circularized CRESS DNA virus genome sequences for in-depth analysis. All 30 CRESS DNA viruses were annotated with replication-associated protein (Rep), and 28 were also annotated with capsid protein (Cap) (Supplementary Table S1). We examined their frequency in 54 virome libraries containing a diverse range of data, including nine time points, seven locations (Qinzhou in Guangxi, Yangjiang in Guangdong, Zhuhai, Huidong, Lianjiang, Shenzhen, and Tanwei), and two tissue types of *Crassostrea hongkongensis* (mixed tissues and hemolymph) [13]. Among these viruses, a total of 12 CRESS DNA-related viruses (YJd1-247829, YJr1-38384, YJd1-191657, ZHd1-462894, YJr1-250706, YJd1-351511, YJr1-251171, YJr1-16749, YJd1-344351, YJd1-334459, YJd1-374311, YJr1-249903) were detected in at least 10 virome libraries, with total TPM values 10–100 times higher than other CRESS DNA viruses. Notably, YJd1-351511, YJd1-247829, and YJr1-38384 were identified in 15, 25, and 30 virome libraries, respectively, and their total TPM values reached 225.52, 275.34, and 347.97, respectively (Supplementary Table S1). This highlights the widespread presence of CRESS DNA viruses in cultivated oysters, demonstrating high diversity and abundance. These findings may have important implications for the management and control of viral infections in aquatic organisms.

### 3.2. Different Evolutionary Rates of Rep and Cap

**Figure 1:**
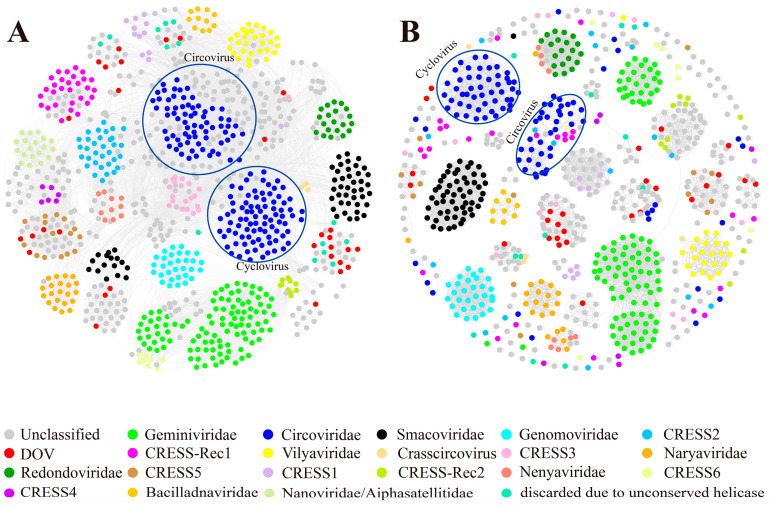
Similarity clustering networks of oyster-associated CRESS DNA virus sequences. (**A**) Clustering network of 1012 Rep protein sequences. (**B**) Clustering network of 827 capsid protein sequences. The networks were visualized using the Fruchterman–Reingold algorithm in Gephi (version 0.9.2). Dots represent different proteins. Edges indicate that the DIAMOND BLASTP scores ≥ 150 (**A**) and ≥42.7 (**B**) between the connected dots.

Constructing a reliable phylogenetic tree can be challenging due to variations in virus sequence similarity. To address this, we compiled a comprehensive dataset comprising all 30 oyster-derived CRESS DNA virus genome sequences along with high-similarity viruses from the NR database and those used in Kazlauskas’s study [31]. We created a similarity network for Rep and Cap based on their protein sequence pairwise identity (Figure 1). The network includes 1012 Rep sequences and 827 Cap sequences. In Figure 1A, the DOV Reps (red dots) are approximately categorized into nine groups, including CRESS5, *Smacoviridae*, *Circovidae*, CRESS-Rec1, and five unclassified groups. Similarly, in Figure 1B, the DOV Caps (red dots) are roughly grouped into 10 clusters, including CRESS5, CRESS-Rec2, and eight unclassified groups. Interestingly, two Caps from DOV form a distinctive group that shares no similarity with other capsid proteins.

Upon examining the clustering results depicted in Figure 1, it is evident that Rep and Cap display noticeable distinctions. Only *Redondoviridae*, *Genomoviridae*, *Bacilladnaviridae*, and *Vilyaviridae* exhibit entirely consistent clustering, implying that the majority of Rep and Cap groups exhibit partially divergent evolutionary patterns. Despite the overall consistent clustering of *Circoviridae*, certain Caps are scattered beyond the primary clustering group of *Circoviridae* (Figure 1B). While the number of corresponding clusters in the Rep and Cap network graphs for *Geminiviridae*, *Smacoviridae*, *Naryaviridae*, and *Nenyaviridae* differs (usually due to differences at the genus level), the overall clustering patterns at the family level are consistent. However, it has been observed that in the majority of CRESS clusters, such as CRESS1-6 and CRESS-Rec1, 2 [31], Rep exhibits good clustering, whereas Cap proteins are mostly unable to aggregate and are dispersed at various positions in the clustering network. Therefore, it is imperative to not solely rely on Rep for the systematic classification of CRESS DNA viruses; Cap, as another critical protein, must also be adequately considered.

### 3.3. The Prevalence of Genetic Recombination in CRESS DNA Viruses

**Figure 2:**
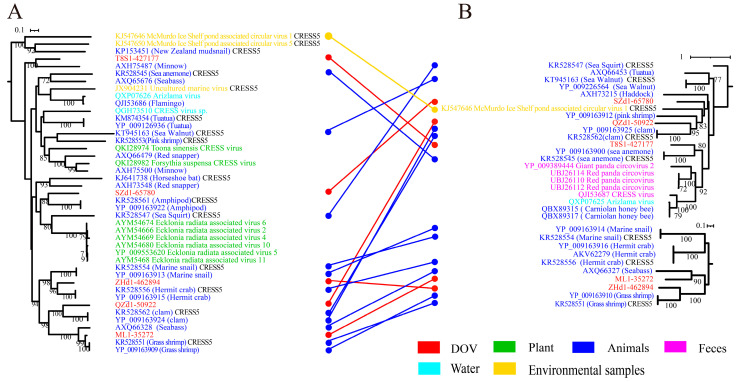
Maximum likelihood phylogenetic tree of CRESS5 and oyster-related viruses. The maximum likelihood phylogenetic tree was constructed using IQtree (version 2.1.4) based on the Rep (**A**) and Cap (**B**) amino acid sequences of CRESS5. ModelFinder was set to MFP, and 1000 ultrafast bootstraps were used. Bootstrap values > 70 are shown. The color of the line corresponds to the color of the virus source shown. Extensive recombination can be inferred when replicase proteins are associated with coat proteins and with different types of structural proteins and vice versa.

To enhance the delineation of the classification between oyster CRESS DNA viruses and other members of related virus families, we constructed maximum likelihood phylogenetic trees based on Reps and Caps for each cluster that encompasses DOV sequences, as shown in Figure 1. These trees provide a visual representation of the evolutionary relatedness of the viruses under study and can aid in understanding the genetic diversity and evolutionary history of these viruses. Among them, five oyster CRESS DNA viruses are grouped in the CRESS5 clade, and these viruses from oysters are most closely related to the animal source branch (highlighted in blue) (Figure 2A). Overall, the branches of the phylogenetic tree of the two proteins correspond well. Interestingly, the Rep and Cap branches of QZd1-50922 consistently show the strongest association with the circular virus (YP_009163924) linked to clams, despite spanning two branches within the CRESS5 clade as the only recombinant viruses. According to research findings, it appears that these particular recombinant viruses may have infected the bivalve ancestors before the separation of oyster and clam species or have a wide host range (including oysters and clams). It is noteworthy that the Rep of ZHd1-462894 shows a stronger correlation to the circular virus discovered in hermit crabs, whereas its Cap is more closely linked to a virus found in grass shrimp, both of which are crustaceans. This indicates that these viruses may undergo recombination during transmission between different crustacean hosts. According to analysis, the Rep of ML1-35272 shares a striking similarity with the circular virus found in shrimp, with an AAI (average amino acid sequence identity) of 93% and ANI (average nucleotide identity) of 91.2%. On the other hand, the Rep of T8S1-427177 appears to have a closer relationship with the circular virus found in minnows (AXH75487). However, its Cap exhibits a higher degree of resemblance with a virus linked to invertebrate sea anemones (YP_009163900), with an AAI of 39.41%. These findings suggest that the predecessors of this viral strain could have a more diverse host range, leading to the possibility of recombination between different virus ORFs.

**Figure 3:**
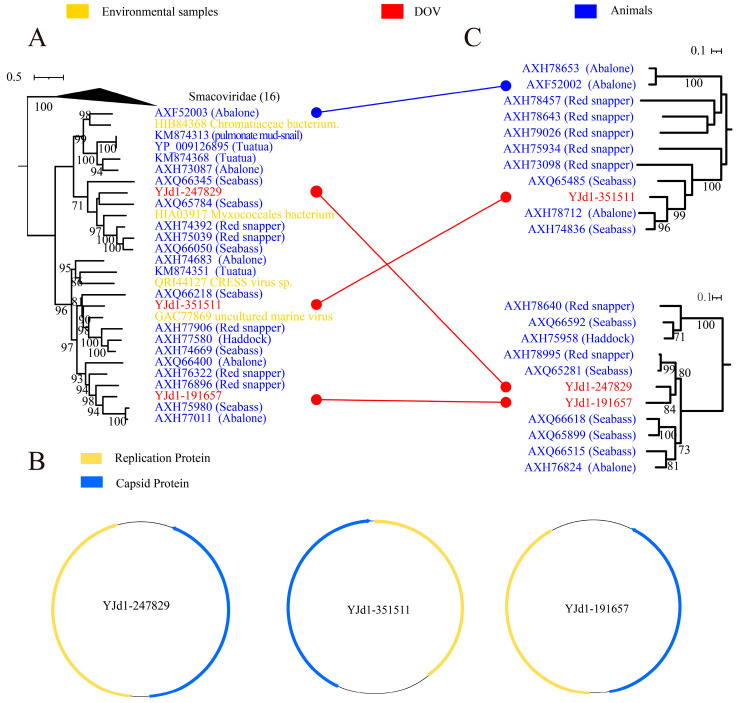
Maximum likelihood phylogenetic tree (**A**,**C**) and genome structure (**B**) of *Smacoviridae* and oyster-related viruses. The maximum likelihood phylogenetic tree was constructed using IQtree (version 2.1.4) based on the Rep amino acid sequences of Smacoviridae-like viruses. ModelFinder was set to MFP, and 1000 ultrafast bootstraps were used. Bootstrap values > 70 are shown. The color of the line corresponds to the color of the virus source shown. Extensive recombination can be inferred when replicase proteins are associated with coat proteins and with different types of structural proteins and vice versa. Genome structure was used for SnapGene (version 4.3.6).

*Smacoviridae* falls under *Cressdnaviricota*, *Arfviricetes*, and *Cremevirales* and is classified as a CRESS DNA virus with ambisense orientation ORFs. It is commonly detected in fecal samples obtained from a variety of animals, including humans, vertebrates, and dragonflies. Furthermore, it has been identified in livestock serum as well as tracheal aspirates and insect samples [40,41]. Our research has revealed that three viruses found in oysters share a close relationship with *Smacoviridae*, as shown in Figure 1A and Figure 3A. The Rep and Cap genes of these viruses are also ambisense (Figure 3B). The Rep phylogenetic tree in Figure 3A indicates that these oyster-derived viruses (YJd1-247829, YJd1-351511, and YJd1-191657) are closely related to viruses from animal sources, forming a distinct branch independent of *Smacoviridae*. Additionally, the capsid protein of these viruses does not exhibit any similarity to *Smacoviridae* (Figure 3C). Based on these findings, we can conclude that these three viruses and their respective groups represent a novel category that is related to *Smacoviridae* but markedly different. In addition, this particular group of viruses primarily consists of strains found in aquatic creatures like fish and mollusks (Figure 3A,C with the blue branches). Notably, Reps of YJd1-191657 and YJd1-247829 exhibit no resemblance to each other but are most closely related to viruses found in abalone and seabass, as evidenced by AAI scores of 52.78% and 62.45%, respectively. Although Caps of YJd1-191657 and YJd1-247829 share significant similarities (with an AAI of 60%), they still cluster with capsid proteins from viruses found in abalone, seabass, and red snapper. This suggests that they are likely related to marine animals and provides substantial evidence for the evolutionary paths of CRESS DNA viruses’ Rep and Cap differences or potential recombination events.

**Figure 4:**
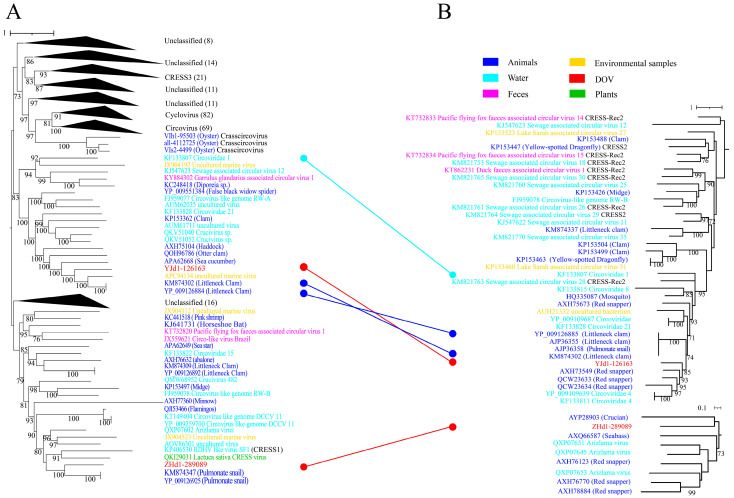
Maximum likelihood phylogenetic tree of oyster-related *Cirlivirales*. The maximum likelihood phylogenetic tree was constructed using IQtree (version 2.1.4) based on the Rep (**A**) and Cap (**B**) amino acid sequences of CRESS DNA viruses. ModelFinder was set to MFP, and 1000 ultrafast bootstraps were used. Bootstrap values > 70 are shown. The color of the line corresponds to the color of the virus source shown. Extensive recombination can be inferred when replicase proteins are associated with capsid proteins and with different types of structural proteins and vice versa.

*Cirlivirales*, as proposed by Krupovic, encompasses both the *Circoviridae* and CRESS1-3 groups [17]. Figure 1A illustrates a significant cluster that comprises *Circoviridae* and CRESS3, aligning with the parameters defined for *Cirlivirales*. Notably, the cluster is primarily dominated by *Circoviridae* and encompasses the *Crasscircovirus* identified by Jiang et al. [13] as well as two oyster-associated CRESS DNA viruses discovered in this study. It is worth mentioning that the results of the Rep and Cap phylogenetic analyses (Figure 4) indicate that the two oyster-associated CRESS DNA viruses discovered in this study belong to two distinct branches that are independent of the previously identified groups. These branch viruses predominantly originate from aquatic environments or aquatic animals, with only a few coming from plants or terrestrial animals. This suggests that the viruses in these branches may potentially infect aquatic eukaryotes. Additionally, evidence of ORF recombination was still found in the evolution tree of *Cirlivirales*. For instance, the Rep of ZHd1-289089 was found to be more closely related to pulmonate snails, while its Cap was closer to viruses found in fish (crucian).

**Figure 5:**
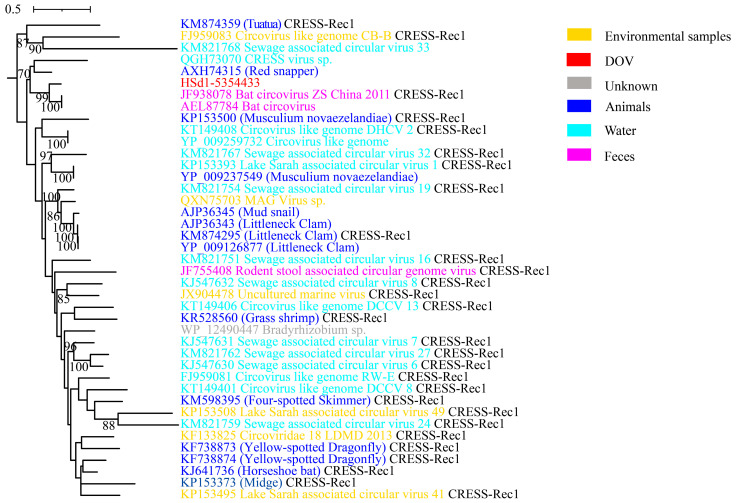
Maximum likelihood phylogenetic tree of CRESS-Rec1 and oyster-related virus. The maximum likelihood phylogenetic tree was constructed using IQtree (version 2.1.4) based on the Rep amino acid sequences of CRESS-Rec1. ModelFinder was set to MFP, and 1000 ultrafast bootstraps were used. Bootstrap values > 70 are shown.

CRESS DNA viruses demonstrate a high degree of recombination both in Rep and Cap, with significant recombination also taking place within the Rep. Extensive research has revealed that approximately 71% of Rep’s HUH (HUH endonucleases, histidine-hydrophobic-histidine) and S3H (Superfamily 3 Helicase) domains manifest distinct evolutionary histories [23]. Among them, the Rep of CRESS-Rec1 is believed to be composed of smacovirus-like HUH and circovirus-like S3H domains. Of the 30 viruses analyzed, only two were not annotated to Caps; HSd1-5354433, which clustered with CRESS-Rec1, was one of them (Figure 1A). In the maximum likelihood phylogenetic tree of Rep for CRESS-Rec1 (Figure 5), it was surprising to note that HSd1-5354433 had a close association with viruses from terrestrial animals (bats) (JF938078, AEL87784) and had a Rep AAI of 82.40%. This finding implies that CRESS DNA viruses might have the capacity to infect both aquatic and terrestrial animal hosts.

### 3.4. A Large Number of Unclassified CRESS DNA Viruses in Oysters from Yangjiang, China

**Figure 6:**
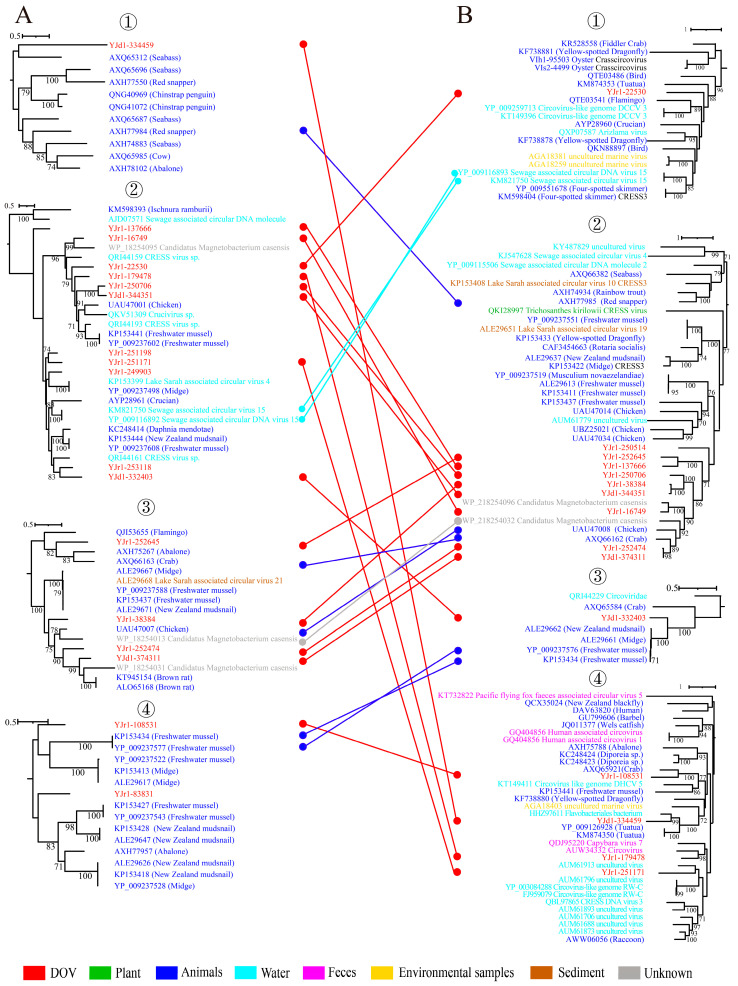
Genetic exchange among CRESS DNA Viruses. Comparison of the phylogenetic trees of replicase-associated proteins (**A**) and capsid proteins (**B**) from four different clusters. ①, ②, ③, and ④ in (**A**) correspond to the four Clusters in Supplementary Figure S2A. And ①, ②, ③, and ④ in (**B**) correspond to the four Clusters in Supplementary Figure S2B. The color of the line corresponds to the color of the virus source shown. Widespread recombination can be inferred when Rep clades are associated with different types of structural protein and vice versa.

The preceding segment explored the viruses associated with oysters that group with recognized viral classifications. Nonetheless, out of the 30 viruses examined in this investigation, up to 18 viruses exhibit connections with categorized groups so remote that they could not be classified (Figure 6 and Supplementary Figure S1). These viruses were all sourced from transcriptomic libraries amplified by phi29, indicating that these ssDNA viruses may share more similarities with RNA viruses. Although these viruses prefixed with YJr were initially assembled from RNA libraries, they were also detected in DNA libraries, but with higher abundance in the RNA libraries (Supplementary Table S2). This may be attributed to their active RNA stage, which makes them detectable in RNA products.

The findings presented in Figure 1 indicate that the Rep and Cap of 18 unclassified CRESS DNA viruses can be grouped into four distinct clusters. Further analysis of each cluster’s phylogenetic tree highlights a significant amount of recombination among these viruses, particularly in phylogenetic trees of Rep② and ③ and Cap②. According to Figure 6, these branches predominantly contain viruses derived from oysters. This finding aligns with the frequently observed recombination phenomenon in CRESS DNA viruses, suggesting that oysters are not only a hub for CRESS DNA viruses’ existence but also their recombination. Generally, viruses that are closely related to oyster viruses originate from aquatic animals or water environments. This fact is exemplified in the evolutionary lineage of Rep, which includes YJd1-334459, YJr1-249903, YJr1-253118, YJd1-332403, YJr1-252645, YJr1-108531, and YJr1-83831. While there are some viruses from terrestrial animals, like rats, chickens, and cows, present in the phylogenetic tree, water environments primarily act as a significant hindrance in preventing the spread of viruses that originated on land. Finally, among the 15 transcriptomic data analyzed, a notable finding is that viruses carrying the prefix YJr belong to the unclassified virus group. This group constitutes the vast majority of the aforementioned cluster, with only four viruses that were discovered through metagenomic sequencing being classified as YJd. Especially in the Rep and Cap② branches, viruses of YJr even form an independent branch distinct from other viruses. This suggests that a group of CRESS DNA viruses specific to oysters have been discovered. Of course, more evidence is needed.

## 4. Discussion

As per the classification criteria set by the International Committee on Taxonomy of Viruses (ICTV), it is expected that members of the same *Circoviridae* species should display more than 80% average nucleotide identity throughout their genomes [42]. This study has revealed that the viral genomes that we analyzed bear resemblance to recognized CRESS DNA viruses, but none of them meet the 80% threshold for average nucleotide identity. Although one virus (ML1-35272) exhibits genome similarity with previously reported circoviruses exceeding 80%, we cannot consider them the same species due to the differences in their capsid protein similarities and low query coverage (Supplementary Figure S2). Therefore, we classified the 30 viruses identified in our study as novel CRESS DNA virus species, which significantly expands the diversity and types of CRESS DNA viruses. On the other hand, Rep has significantly more network connectivity than Cap (Figure 1), suggesting that more Rep is conserved. Rep, as an essential replication-associated protein of viruses, is slower to mutate, which facilitates a unified classification of CRESS DNA viruses. However, the network of Cap is less connected, and Caps evolve faster. As a capsid protein determines the infectivity and host range of viruses, frequent mutation and recombination of Caps may be important to enhance the ecological adaptability and extend the host range of CRESS DNA viruses. Thus, capsid protein should also be used as a basis for viral taxonomics [43]. Generally, the results of Cap clustering and Rep clustering are inconsistent or even contradictory, making it impossible to accurately classify the viruses identified in this study. There are huge differences between the evolution of viruses and that of cellular organisms (including prokaryotes and eukaryotes). For example, CRESS DNA viruses are driven by events such as genome recombination, while cellular organisms are driven by sexual reproduction and chromosomal rearrangements. Thus, the evolution and classification of viruses, such as CRESS DNA viruses, still require a more sophisticated methodology.

In recent times, metagenomic studies have discovered a plethora of CRESS DNA viruses with diverse characteristics across various environmental samples, including the marine environment [44,45] and mollusks, such as *Austrovenus stutchburyi*, *Paphies subtriangulata*, *Dreissena rostriformis bugensis*, and *Amphibola crenata* [20,21]. Our analysis of Rep and Cap through phylogenetic trees of CRESS DNA viruses indicates that most oyster-related CRESS DNA viruses are related to marine environment viruses. They especially share a closer phylogenetic relationship with marine animals or the CRESS DNA viruses discovered in marine environments. It is worth noting that all known hosts of *Circoviridae* within the animal kingdom are bilaterally symmetrical animals, with the possible exception of archaea as hosts for Smacoviridae. Additionally, other known hosts of CRESS DNA include animals, plants, and eukaryotic microorganisms. Therefore, it is inferred that the hosts of the CRESS DNA viruses linked to oysters may include not only oysters but also other marine creatures that come into contact with oysters as well as particular eukaryotic microbes or archaea within oysters [15,46]. To date, there have been no definitive reports of aquatic animals being infected with circoviruses. Circoviruses belonging to the *Circoviridae* with complete genomes have only been discovered in fish [13,47,48,49]. Furthermore, replication-related protein genes of circoviruses have been detected in various fish species, such as European perch, round goby, Indian Labeo rohita, and catla fish, suggesting the prevalence of circoviruses in fish species [33]. Metagenomic techniques have led to the discovery of a noteworthy amount of CRESS DNA viruses in invertebrates like cnidarians, crustaceans, and gastropods [15]. To accurately identify the hosts of these aquatic CRESS DNA viruses, thorough determination methods, such as wet experiments involving isolation and cultivation, artificial infection, electron microscopy observation, and in situ hybridization, are still required.

Furthermore, Rosario delved into the exploration of 21 different marine invertebrates, which encompassed marine snails, anemones, sea squirts, and multiple types of crabs [15]. Through this investigation, a significant number of CRESS DNA viruses were compiled from crustaceans. These findings emphasized the importance of continued research into viruses linked with prevalent marine invertebrates as well as the need for experiments to evaluate any potential ecological effects on these organisms [13]. The topic of filter feeding, especially in the context of densely clustered intertidal organisms such as oysters, has been a subject of significant interest in recent years [13,14,50]. The efficient filter-feeding mechanisms of these organisms, coupled with their group aggregation effects, have been found to have far-reaching implications for the transmission, recombination, and outbreaks of marine viruses across different species [51,52]. The study of these phenomena has the potential to provide valuable insights into the complex ecological dynamics of marine ecosystems and their associated viral populations. As found in this study, the average nucleotide identity (ANI) of ML1-35272 with grass shrimp virus (NC_027786) Rep was 91.2%, and the average amino acid identity (AAI) of HSd1-5354433 with bat virus (JF938078, AEL87784) Rep was 82.40%. These results imply that oysters could be a site of viral recombination, but only for viruses that can infect them, and a critical location for their recombination dissemination into marine and even terrestrial environments [53,54].

## 5. Conclusions

This study delved into the evolution of CRESS DNA viruses in oysters using metagenomic data. The research revealed that oysters contain a significant amount of CRESS DNA viruses, including members of the CRESS5, *Smacoviridae*, *Circovidae*, and CRESS-Rec1 groups, with the majority remaining unidentified. Despite being unclassified, these viruses are plentiful, and the transcriptomic libraries amplified by phi29 indicate the potential transcriptional activity of their ssDNA viruses. The study also identified extensive genetic recombination in CRESS DNA viruses. The Rep of viruses in oysters are more similar to the Rep of eukaryotic viruses, determining their conditions for survival in oysters. Additionally, oyster viruses in Cap are predominantly similar to those found in aquatic animals and exhibit a degree of clustering, implying their potential to infect oysters. CRESS DNA viruses derived from oysters exist mainly in distinct branches, consistent with the observed recombination of CRESS DNA viruses. This finding suggests that oysters may be a hotspot for CRESS virus existence and recombination.

## Data Availability

The data set supporting the results of this article has been deposited in the Genome Sequence Archive (GSA) under BioProject accession code PRJCA007058 [https://ngdc.cncb.ac.cn/gsub/submit/bioproject/subPRO010366/overview] (accessed on 15 August 2022). All viral genomes obtained in this study were deposited in GenBase of the China National Center for Bioinformation (CNCB) with the accession number C_AA056653-82.

## Supplementary Materials

The following supporting information can be downloaded at: https://www.mdpi.com/article/10.3390/v16020228/s1, Table S1: Genomic sequence information of oyster-associated CRESS DNA viruses. Table S2: Analysis of abundance of oyster-associated CRESS DNA viruses. Figure S1: Similarity clustering networks of oyster-associated CRESS DNA virus sequences. (A) Clustering network of 1012 Rep protein sequences. Clusters ①, ②, ③, and ④ correspond to phylogenetic trees in Figure 6A. (B) Clustering network of 827 capsid protein sequences. Clusters ①, ②, ③, and ④ correspond to phylogenetic trees in Figure 6B. The networks were visualized using the Fruchterman–Reingold algorithm in Gephi (version 0.9.2). Dots represent different proteins. Edges indicate DIAMOND BLASTP scores ≥ 150 (A) and ≥42.7 (B) between the connected dots. Figure S2: Genome average nucleotide identity heatmap. Vertical and horizontal axes are sequence IDs of viruses. Parentheses represent the sequence ID of proteins that are closely related to the proteins of CRESS DNA viruses in this study.
