# Supplementary material for: Unveiling CRESS DNA Virus Diversity in Oysters by Virome
Source: Viruses. 2024 Jan 31;16(2):228. doi: 10.3390/v16020228 (PMC10892194; doi:10.3390/v16020228)
Supplement: Supplementary file 1 [file viruses-16-00228-s001.zip › Supplementary Figure S1 Similarity clustering networks of oyster-associated CRESS DNA virus sequences.pdf]

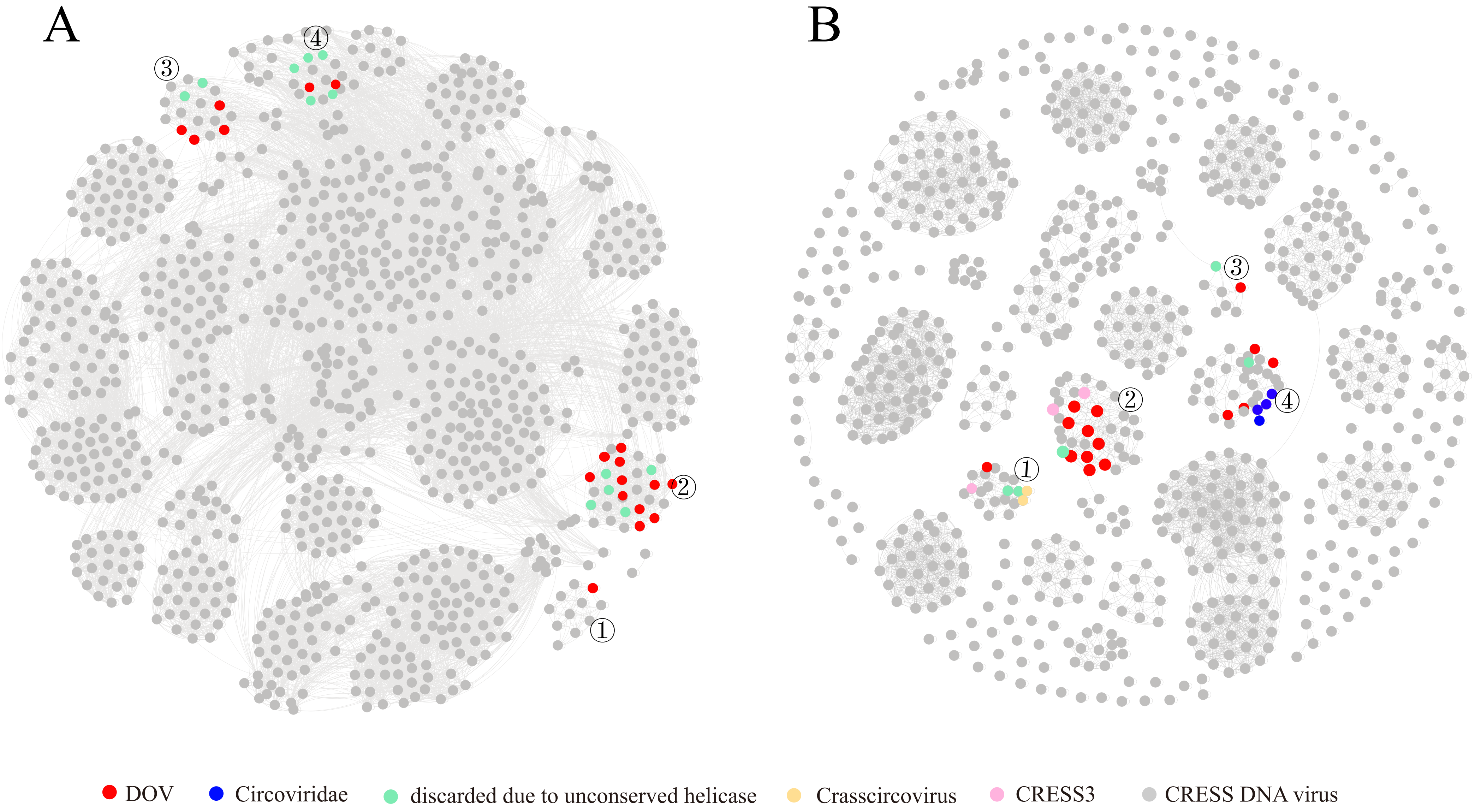
A
B
①
②
③
④
DOV
Circoviridae
discarded due to unconserved helicase
Crasscircovirus
CRESS3
CRESS DNA virus
