# Supplementary material for: Unveiling CRESS DNA Virus Diversity in Oysters by Virome
Source: Viruses. 2024 Jan 31;16(2):228. doi: 10.3390/v16020228 (PMC10892194; doi:10.3390/v16020228)
Supplement: Supplementary file 1 [file viruses-16-00228-s001.zip › Supplementary Figure S2. Genome average nucleotide identity heatmap.pdf]

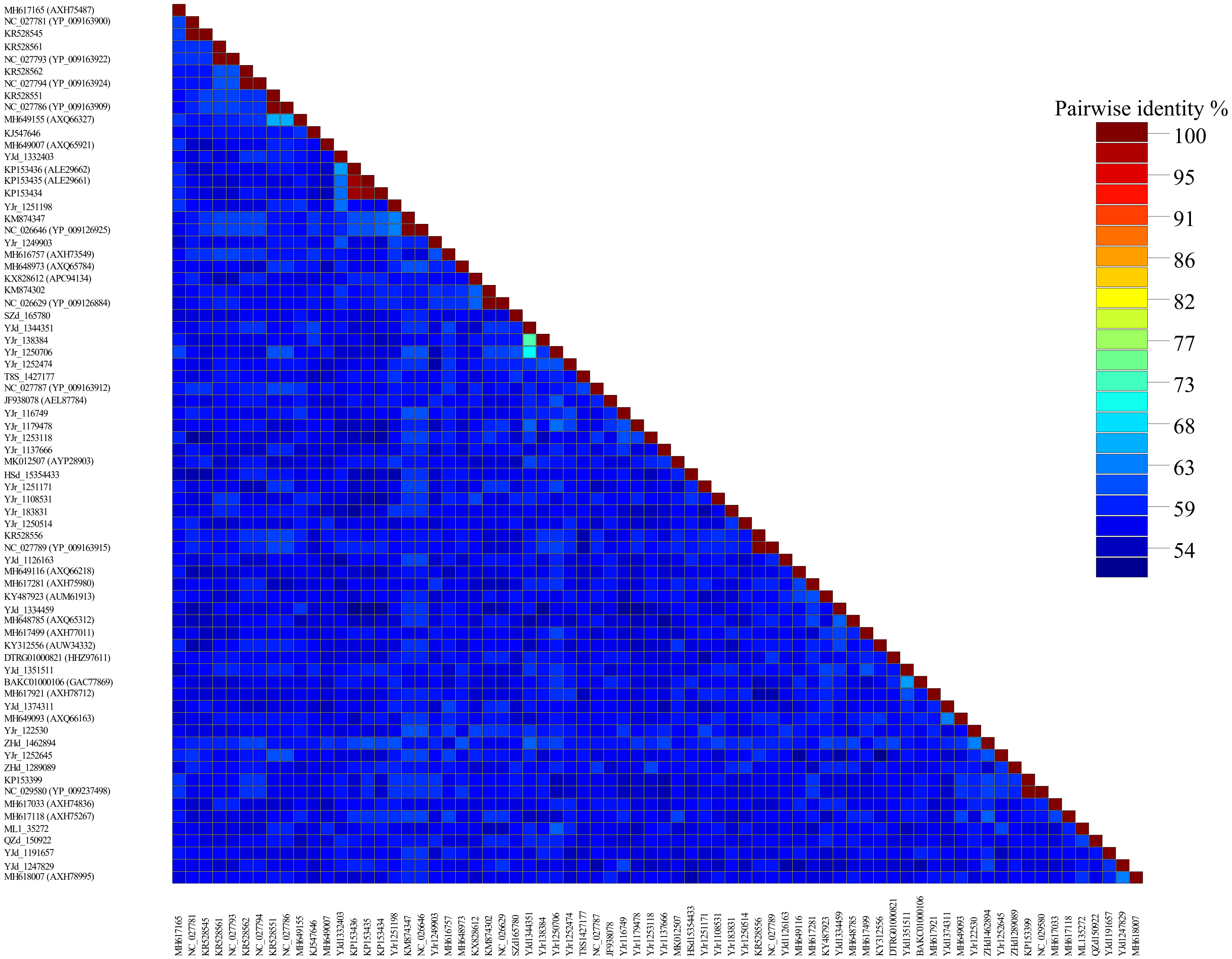
Pairwise identity %
100
95
91
86
82
77
73
68
63
59
54
MH617165 (AXH75487)
NC_027781 (YP_009163900)
KR528545
KR528561
NC_027793 (YP_009163922)
KR528562
NC_027794 (YP_009163924)
KR528551
NC_027786 (YP_009163909)
MH649155 (AXQ66327)
KJ547646
MH649007 (AXQ65921)
YJd_1332403
KP153436 (ALE29662)
KP153435 (ALE29661)
KP153434
YJr_1251198
KM874347
NC_026646 (YP_009126925)
YJr_1249903
MH616757 (AXH73549)
MH648973 (AXQ65784)
KX828612 (APC94134)
KM874302
NC_026629 (YP_009126884)
SZd_165780
YJd_1344351
YJr_138384
YJr_1250706
YJr_1252474
T8S_1427177
NC_027787 (YP_009163912)
JF938078 (AEL87784)
YJr_116749
YJr_1179478
YJr_1253118
YJr_1137666
MK012507 (AYP28903)
HSd_15354433
YJr_1251171
YJr_1108531
YJr_183831
YJr_1250514
KR528556
NC_027789 (YP_009163915)
YJd_1126163
MH649116 (AXQ66218)
MH617281 (AXH75980)
KY487923 (AUM61913)
YJd_1334459
MH648785 (AXQ65312)
MH617499 (AXH77011)
KY312556 (AUW34332)
DTRG01000821 (HHZ97611)
YJd_1351511
BAKC01000106 (GAC77869)
MH617921 (AXH78712)
YJd_1374311
MH649093 (AXQ66163)
YJr_122530
ZHd_1462894
YJr_1252645
ZHd_1289089
KP153399
NC_029580 (YP_009237498)
MH617033 (AXH74836)
MH617118 (AXH75267)
ML1_35272
QZd_150922
YJd_1191657
YJd_1247829
MH618007 (AXH78995)
