# Supplementary material for: Unveiling CRESS DNA Virus Diversity in Oysters by Virome
Source: Viruses. 2024 Jan 31;16(2):228. doi: 10.3390/v16020228 (PMC10892194; doi:10.3390/v16020228)
Supplement: Supplementary file 1 [file viruses-16-00228-s001.zip › Supplementary Table S1 Genomic sequence information of oyster-associated CRESS DNA viruses.pdf]

Supplementary table 1. Genomic sequence information of oyster-associated CRESS DNA viruses

Genome ID	Length	Replication protein	Capsid protein	Total TPM	Detected libraries/Total libraries
HSd1-5354433	1970	1010 .. 1813	-	2.56	9/32
QZd1-50922	2154	1 .. 1092	1115 .. 1942	9.79	4/32
T8S1-427177	2096	1206 .. 2096	157 .. 1029	3.06	6/32
ZHd1-289089	2427	1450 .. 2427	534 .. 1460	15.36	4/32
YJd1-126163	2022	750 .. 1847	2 .. 712	4.81	3/32
SZd1-65780	2084	1169 .. 2059	237 .. 1127	57.63	8/32
ML1-35272	2252	1 .. 909	1008 .. 1997	1.39	7/32
ZHd1-462894	2899	1530 .. 2441	292 .. 1311	167.47	17/32
YJd1-191657	1891	1 .. 750	822 .. 1604	76.55	18/32
YJd1-247829	1995	132 .. 980	1053 .. 1925	275.34	25/32
YJd1-332403	1708	41 .. 577	623 .. 1348	5.32	8/32
YJd1-334459	1702	197 .. 919	965 .. 1702	40.40	11/32
YJd1-344351	1587	653 .. 1384	1 .. 675	133.95	12/32
YJd1-351511	2283	383 .. 1345	1342 .. 2283	225.52	15/32
YJd1-374311	1840	232 .. 1041	1016 .. 1840	13.74	10/32
YJr1-108531	1790	990 .. 1583	1 .. 894	8.17	5/32
YJr1-137666	1881	1 .. 678	1122 ..1907	12.48	2/32
YJr1-16749	1833	906 .. 1691	257 .. 925	177.49	14/32
YJr1-179478	1706	596 .. 1444	7 .. 657	8.76	3/32
YJr1-22530	1631	647 .. 1330	1 .. 669	2.87	3/32
YJr1-249903	1662	315 .. 962	916 .. 1662	7.50	10/32
YJr1-250514	1895	1206 .. 1895	1 .. 840	12.53	7/32

Genome ID	Length	Replication protein	Capsid protein	Total TPM	Detected libraries/Total libraries
YJr1-250706	1438	678 .. 1421	2 .. 739	137.76	16/32
YJr1-251171	1683	806 .. 1489	1 .. 813	8.71	14/32
YJr1-251198	1650	230 .. 850	816 .. 1487	9.58	3/32
YJr1-252474	1708	1 .. 684	659 .. 1468	12.59	7/32
YJr1-252645	1601	146 .. 691	688 .. 1458	10.06	5/32
YJr1-253118	1674	759 .. 1418	-	10.51	6/32
YJr1-38384	1633	680 .. 1447	28 .. 702	347.97	19/32
YJr1-83831	1 760	282 .. 857	1080 .. 1760	7.26	7/32
